# Isoflavone Changes in Immature and Mature Soybeans by Thermal Processing

**DOI:** 10.3390/molecules26247471

**Published:** 2021-12-10

**Authors:** Shanshan Qu, Soon Jae Kwon, Shucheng Duan, You Jin Lim, Seok Hyun Eom

**Affiliations:** 1 Department of Horticultural Biotechnology, College of Life Sciences, Kyung Hee University, Yongin 17104, Korea; qss1996@khu.ac.kr (S.Q.); dsc97@khu.ac.kr (S.D.); yujn0213@khu.ac.kr (Y.J.L.); 2 Advanced Radiation Technology Institute, Korea Atomic Energy Research Institute, Jeongeup 56212, Korea; soonjaekwon@kaeri.re.kr

**Keywords:** isoflavone conversion, thermal process, immature seeds, mature seeds, internal water content

## Abstract

The isoflavone changes occurring in mature soybeans during food processing have been well studied, but less information is available on the changes in immature soybeans during thermal processing. This study aimed to determine the effect of thermal processing by dry- or wet-heating on the changes in the isoflavone profiles of immature and mature soybeans. In the malonylglycoside forms of isoflavone, their deglycosylation was more severe after wet-heating than after dry-heating regardless of the soybean maturity. The malonyl forms of isoflavones in the immature seeds were drastically degraded after a short wet-heating process. In the acetylglycoside forms of isoflavone, dry-heating produced relatively low amounts of the acetyl types in the immature soybeans compared with those in the mature soybeans. These results were explained by the content of acetyldaidzin being relatively less changed after dry-heating immature soybeans but increasing four to five times in the mature soybeans. More of the other types of acetylglycoside were produced by dry-heating soybeans regardless of their maturity. Acetylgenistin in wet-heating was a key molecule because its content was unchanged in the immature soybeans during processing but increased in the mature soybeans. This determined the total acetylglycoside content after wet-heating. In contrast, most of the acetyl forms of isoflavone were produced after 90 to 120 min of dry-heating regardless of the seed maturity. It can be suggested that the pattern of isoflavone conversion was significantly affected by the innate water content of the seeds, with a lower water content in the mature soybeans leading to the greater production of acetyl isoflavones regardless of the processing method even if only applied for a relatively short time. The results suggested that the isoflavone conversion in the immature soybeans mainly follows the wet-heating process and can be promoted in the application of stronger processing.

## 1. Introduction

Soybeans (*Glycine max* L.) are one of the most widely consumed legumes in the world. As well as their main role in providing protein, carbohydrates, and oil, soybeans are also a rich source of phytochemicals, particularly isoflavones [1,2]. The content of isoflavones, a type of flavonoid, is greater in soybeans than in other legumes [3]. The 12 major isoflavones in soybeans can be classified into four main forms: aglycones (daidzein, glycitein, and genistein); β-glycosides (daidzin, glycitin, and genistin); acetylglycosides (acetyldaidzin, acetylglycitin, and acetylgenistin); and malonylglycosides (malonyldaidzin, malonylglycosides, and malonylgenistin) [4,5]. Of these isoflavone groups, malonylglycosides are the predominant form in raw soybeans, followed by β-glycosides and acetylglycosides, with aglycones rarely observed [6]. Epidemiological studies have reported that the presence of different types of isoflavone in soybeans contributes to various biological activities, such as reducing the risk of cancer, cardiovascular disease, and osteoporosis, and relieving menopausal symptoms [7,8,9,10,11,12]. Among the four forms of isoflavones, the bioavailability of malonyl-conjugated isoflavones was lower than that of corresponding non-conjugated β-glycoside isoflavones [13], and that of isoflavone aglycons was highest because aglycones were more easily and quickly absorbed by the intestine [14]. Furthermore, some studies reported that non-conjugated glycoside isoflavones also possessed high-quality antioxidant activity similar to aglycones. Thus, soybeans with a high content of non-conjugated glycosides and aglycones had high-quality antioxidant activity [15].

Because of their grassy-beany flavor and bitter taste [16,17], raw mature soybeans are mainly consumed after thermal processing, such as boiling or roasting, which greatly improves the flavor of the soybeans and soy products [18,19,20]. It has also been reported that thermal processing causes the conversion or degradation of isoflavones [21,22,23,24]. The predominant isoflavone form (malonylglycosides) is usually converted into acetylglycosides and β-glycosides by thermal processing [25,26,27,28,29,30], with the patterns of conversion depending significantly on the severity of heating. In general, the increase in the contents of isoflavone acetylglycosides and β-glycosides occurs through the decarboxylation and deesterification of malonylglycosides, respectively [28,31]. The wet-heating method promotes deesterification more than dry-heating as it causes the rapid conversion of isoflavone malonylglycosides to β-glycosides rather than acetylglycosides [32,33]. Chien et al. [34] reported no changes in the glycosides and aglycones during dry-heating below 150 °C, but Huang and Chou [35] reported a decrease in the aglycones in soybeans steamed for 30 min at temperatures above 60 °C.

The changes in isoflavone content during thermal processing in mature soybeans have been widely reported, but those in immature soybeans have rarely been studied. Immature soybeans, also known as edamame or maodou, are harvested when the green seeds fill the pod and have a water content of 60 to 65% [36,37]. Immature soybeans are also rich in isoflavones. The difference in the isoflavone content between immature and mature soybeans is significantly affected by the cultivar. Simonne et al. [38] investigated the iso-flavone contents of immature and mature beans of five soybean cultivars and found that the immature beans contained twice the isoflavone content of the mature beans in four of the cultivars, whereas Kim et al. [39] reported that the mature soybeans of several cultivars contained more isoflavone than the immature soybeans. Immature soybeans also exhibit a beany off-flavor and unique taste compared with mature soybeans [40]. Traditionally, in East Asia, immature soybeans have also been consumed as vegetables and snacks after thermal processing, such as boiling [40]. Simonne et al. [38] studied the influence of processing methods, such as boiling and freeze-drying, on the distribution of isoflavones in immature soybeans, but the patterns of variation in isoflavone content in immature soybeans during thermal processing are still unclear, particularly compared with those in mature soybeans.

Therefore, the objectives of this study are: to compare the effects of thermal processing (dry- and wet-heating) on variations in the isoflavone profiles of immature and mature soybeans, and to determine the effect of the internal water content of soybeans on the isoflavone content during thermal processing by varying the soaking time of mature seeds before dry-heating. This study will provide basic information on utilizing isoflavones in soybeans with different levels of maturity.

## 2. Results and Discussion

### 2.1. Physiological Characteristics of Immature and Mature Soybeans

**Figure 1:**
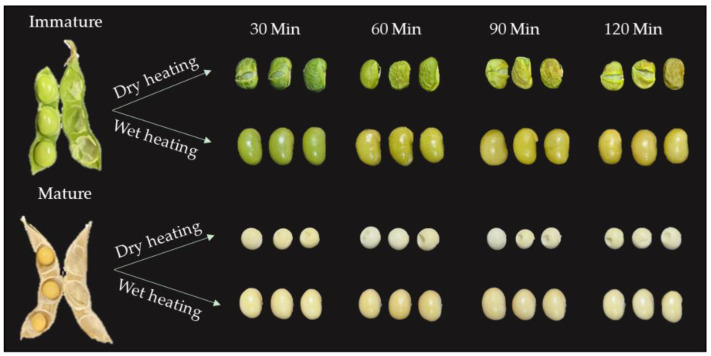
Morphologies of immature and mature soybeans after dry- and wet-heating for different times.

The physical characteristics of the raw immature and mature soybeans are shown in Table 1. The water contents of the immature and mature seeds were 66.87% and 13.04%, respectively. Takahashi et al. [37] reported that, in immature soybeans, the water contents of four different cultivars ranged from 61.5% to 76.8%, similar levels to those of the present study. It has also been reported that the moisture content of mature soybeans was 10–15%, a similar content to the present study [41,42]. The dry weight of 10 immature seeds (0.66 g) was significantly lower than that of 10 mature seeds (0.88 g) (Table 1). As expected, a remarkable difference in the SSC of the immature (2.75 °Brix) and mature seeds (15.76 °Brix) was also observed (Table 1). Sale and Campbell [43] reported that a dramatic reduction in the water content of soybeans was accompanied by a steady accumulation of dry matter and SSC during seed maturation from the R6 (immature) to the R8 (mature) stages. Figure 1 shows the morphologies of the immature and mature soybeans before and after thermal processing. After dry-heating, the immature and mature soybeans had shrunk, particularly the immature soybeans, whereas they had both swelled after wet-heating, particularly the mature seeds. A significant variation in seed color (green to yellow) was also observed, particularly for the immature soybeans, possibly because of the Maillard reaction [44].

### 2.2. The Variation in the Total Isoflavone Content and Four Isoflavone Forms of Soybeans during Thermal Processing

**Figure 2:**
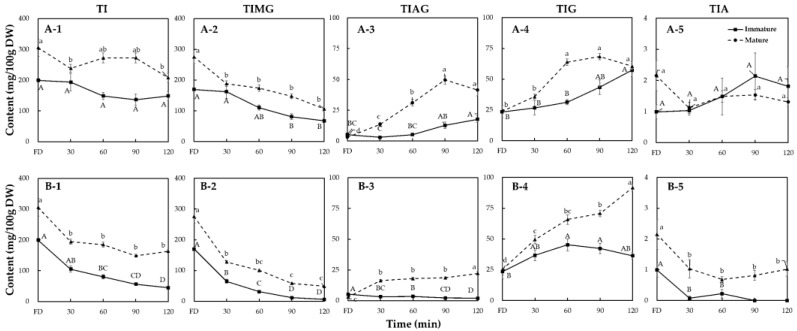
Changes in the total isoflavone (TI), total isoflavone malonylglycosides (TIMG), total isoflavone acetylglycosides (TIAG), total isoflavone β-glycosides (TIG), and total isoflavone aglycones (TIA) contents of immature and mature soybeans during dry-heating (**A**) and wet-heating (**B**). Data are shown as mean ± SE (*n* = 3). Different letters indicate significant differences at *p* < 0.05 by Tukey’s studentized range (HSD) test.

Many studies have reported the effect of thermal processing on the isoflavone profiles of mature soybeans or soy products [25,28,30,32]. However, studies comparing the thermal transformation or degradation of isoflavones in soybeans at different maturity levels are still limited. The variations in the total isoflavone content (TI) and in the other four forms of isoflavone (total isoflavone malonylglycosides (TIMG); total isoflavone acetylglycosides (TIAG); total isoflavone β-glycosides (TIG); and total isoflavone aglycones (TIA)) in immature and mature soybeans during thermal processing are shown in Figure 2.

Before thermal treatment (freeze-dried, FD), the TI of the mature soybeans (304.51 mg/100 g DW) was 1.6 times higher than that of the immature soybeans. The isoflavone form with the highest content in both the immature and mature soybeans was TIMG, followed by TIG, with small amounts of TIAG and TIA (Figure 2). These results were consistent with those of Kim et al. [39], who reported that the TI of mature soybeans was higher than that of immature soybeans, with malonylglycoside isoflavones being the predominant form in both immature and mature soybeans, followed by the β-glycoside, acetylglycoside, and aglycone isoflavone forms.

After thermal processing, the TI content tended to decrease regardless of the seed maturity and processing method. The TI content decreased significantly after wet-heating: in mature soybeans from 304.51 to 163.49 mg/100 g DW; in immature soybeans from 199.34 to 44.63 mg/100 g DW; compared with dry-heating: in mature soybeans to 200.32 mg/100 g DW; and in immature soybeans to 147.15 mg/100 g DW). This may have been caused by the difference in humidity between wet- and dry-heating affecting the transfer of thermal energy [34,45], which accelerates the decrease in TI content. This decrease in the TI content of thermally treated soybeans or soy products has been widely reported [13,29,38]. However, the present study has been the first to compare changes in TI content in soybeans at different maturity levels and after different processing methods. The significant decrease in the TIMG content was considered to be the main reason for the reduction in the TI content (Figure 2). As mentioned previously, malonylglycoside isoflavones are the predominant form in soybeans. Similar to the TI content, the content of TIMG in both the immature and mature soybeans tended to decrease after thermal treatment, and soybeans with a higher internal water content under wet-heating conditions promoted this decreasing trend (Figure 2(A-2,B-2)). The relative thermal instability of malonyl-conjugated β-glycoside isoflavones has been reported previously [34]. Wet-heating degraded malonyl isoflavones more than dry-heating because of the higher moisture content in wet-heat process [32,45].

**Figure 3:**
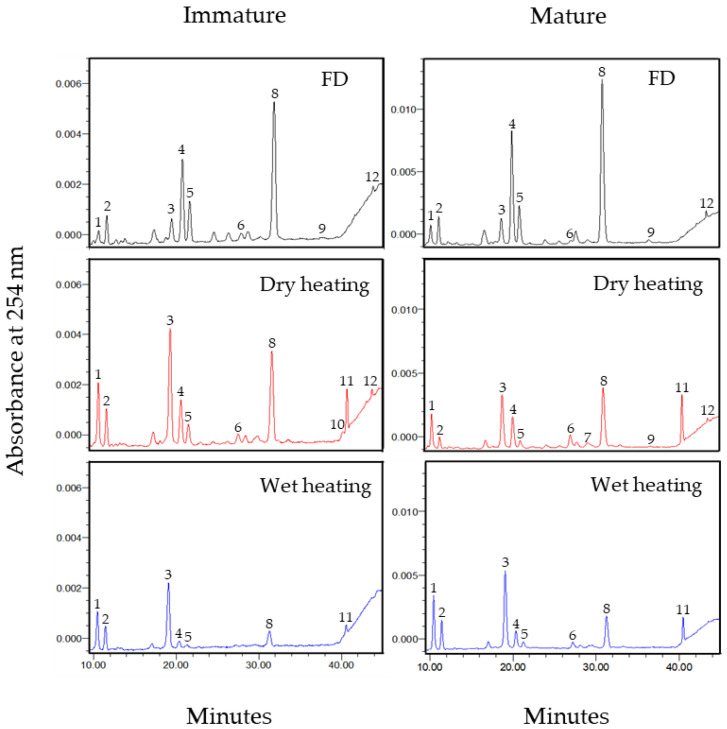
Twelve isoflavones of immature and mature soybean seeds after 120 min of dry- and wet-heat treatment determined by HPLC. 1, daidzin (DZI); 2, glycitin (GLI); 3, genistin (GNI); 4, malonyldaidzin (MDZI); 5, malonylglycitin (MGLI); 6, acetyldaidzin (ADZI); 7, acetylglycitin (AGLI); 8, malonylgenistin (MGNI); 9, daidzein (DZE); 10, glycitein (GNE); 11, acetylgenistin (AGNI); 12, genistein (GNE).

The decrease in the TIMG content, usually accompanied by an increase in the TIAG content in soybeans processed by thermal treatments, such as roasting [46] and baking [47], or in processed soy products, such as cooked soybeans [48] and soy milk [31], has been widely investigated. The increase in the TIAG content was caused by the decarboxylation of malonyl isoflavones during heat processing. Similarly, in mature soybeans, significant increases in the TIAG content after thermal processing were also observed (Figure 2(A-3,B-3)), with dry-heating increasing its content from 3.09 to 41.47 mg/100 g DW, thus being more effective than wet-heating, which increased its content from 3.09 to 22.23 mg/100 g DW. The difference in the effect of dry- and wet-heating on TIAG content can be explained by the maximum degradation rate of malonyl to acetyl isoflavones under the dry-heat condition, while a low conversion rate of malonyl to acetyl isoflavones was found under the wet-heat condition [34]. It indicated that wet-heating tended to convert or degrade malonylglycosides to other isoflavone derivatives instead of acetylglycosides, while the decarboxylation of malonyl isoflavones also occurred simultaneously. However, immature soybeans showed the reverse behavior in response to dry- and wet-heating: like the mature soybeans, dry-heating increased the TIAG content of immature beans from 5.20 to 19.36 mg/100 g DW, whereas wet-heating significantly decreased the TIAG content from 5.20 to 2.04 mg/100 g DW. As there has been no comparative study on the variation in the TIAG content in immature soybeans under dry- and wet-heating conditions, the present study is the first to report that the variation in the TIAG content of immature soybeans after thermal processing is different from that of mature beans. This may have been caused by the higher internal water content of immature soybeans significantly promoting the transfer of energy under wet-heating compared with dry-heating conditions. This led to the rapid conversion of acetylglycosides to β-glycosides, or the degradation of acetylglycosides [34], a speculation also supported by Huang and Chou [35], who reported that the acetyl isoflavones content of soaked black soybeans decreased after steaming at 100 °C for 30 min. Under dry-heat conditions, the TIAG content of the mature soybeans increased significantly compared with the immature soybeans (Figure 2(A-3)). This could be interpreted as the variation in the TIAG content of immature soybeans with their higher internal water content under dry-heating being similar to that of mature soybeans under wet-heating.

**Figure 4:**
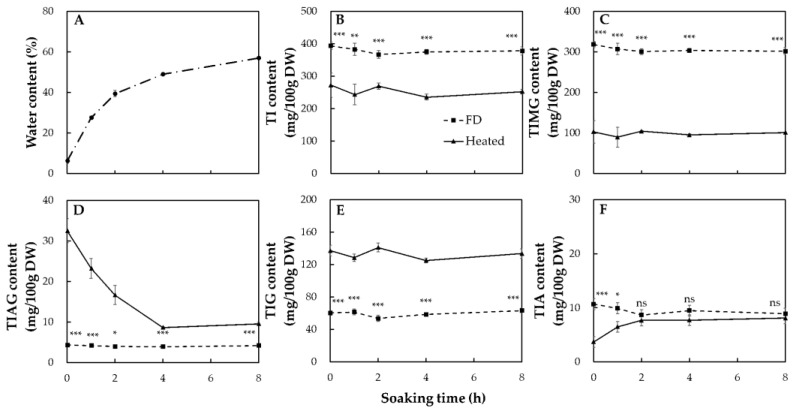
Water content of mature seeds during serial soaking (**A**), and isoflavone contents in soaked seeds after freeze-drying (FD) and dry-heating (**B**–**F**). (**A**), water content; (**B**), total isoflavone (TI); (**C**), total isoflavone malonylglycosides (TIMG); (**D**), total isoflavone acetylglycosides (TIAG); (**E**), total isoflavone β-glycosides; (**F**), total isoflavone aglycones. Data are shown as mean with standard error (*n* = 3). Asterisks indicate statistically significant differences (*, *p* < 0.05; **, *p* < 0.01; ***, *p* < 0.001; ns indicates no significance).

Thermal processing also increased the TIG content by deesterifying malonylglycoside and acetylglycoside isoflavones [46,47,48,49,50]. The patterns of variation in the TIG content were significantly affected by the processing methods and level of seed maturity (Figure 2(A-4,B-4)). In mature soybeans, the effect of wet-heating, increasing the TIG content from 25.16 to 91.64 mg/100 g DW, was better than that of dry-heating, where it increased from 25.16 to 59.00 mg/100 g DW. This can be partly explained by the deesterification of acetylglycoside isoflavones to form β-glycoside isoflavones under wet-heating (Figure 2(A-3,B-3)). Huang and Chou [35] have also reported that the TIG content of mature soybeans increased as the temperature of the thermal treatment increased. In contrast, the opposite patterns were observed for immature soybeans. The TIG content after dry-heating, ranging from 23.40 to 57.30 mg/100 g DW, was higher than that after wet-heating, which ranged from 23.40 to 45.26 mg/100 g DW. As mentioned earlier, the high internal moisture content of the soybeans under wet-heating may have led to excessive energy transfer, leading to the further conversion or degradation of the β-glycoside isoflavones. Thermal processes, such as oven drying [46], baking [47], frying [48], steaming [35], and autoclaving [50], have been reported as important methods for increasing the TIG content of mature soybeans. However, the effect of dry- and wet-heating on the variation in the TIG content of immature soybeans has not been studied. The results suggest that, unlike mature soybeans, dry-heating is more suitable for processing immature soybeans and leads to a higher TIG content than wet-heating.

As mentioned earlier, TIA accounts for only a small proportion of the TI in soybeans. Figure 2(A-5,B-5) shows the variation in the TIA content at different maturity levels of soybeans after thermal processing. The TIA content remained stable in the mature soybeans during dry-heating but decreased significantly during wet-heating. Similar results on mature soybeans have also been observed by Kao et al. [51] and Aguiar et al. [50] for dry- and wet-heating, respectively. The isoflavone deglycosylation from glycoside form to aglycone was observed only under high temperature, possibly because it was difficult to break down the glycoside groups to form aglycones at relatively low temperatures [52]. The present study has shown that the variation in the TIA content of the immature soybeans was similar to that of mature soybeans. In this study, because we used only one cultivar, further studies using numerous cultivars are required to understand the isoflavone deglycosylation patterns of soybeans regarding whether the isoflavone changes among immature soybean cultivars by thermal processing are presenting the same patterns or not.

### 2.3. Correlation Analysis between Isoflavone Form and Corresponding Individual Isoflavones in Immature and Mature Soybeans

To clarify the patterns of how isoflavones changed for different maturity levels of soybeans during thermal processing, the correlations between the contents of the isoflavone form and corresponding individual isoflavones were analyzed. Table 2 and Table 3 show the variations in the content of 12 individual isoflavones (IMG (MDZI, MGLI, and MGNI), IAG (ADZI, AGLI, and AGNI), IG (DZI, GLI, and GNI), and IA (DZE, GLE, and GNE)) in immature and mature soybeans, respectively, after dry- and wet-heating. The profiles of 12 individual isoflavones detected by the reversed-phase high-performance liquid chromatography (HPLC) in immature and mature soybeans before treatment and after 120 min of thermal treatment are shown in Figure 3.

Of the three types of malonylglycoside isoflavones, MGNI (78.18 mg/100 g DW in immature soybeans; 141.25 mg/100 g DW in mature soybeans) and MDZI (62.32 mg/100 g DW in immature soybeans; 119.20 mg/100 g DW in mature soybeans) were the main isoflavones in the FD samples (Table 2 and Table 3). The deglycosylation of MGNI, MDZI, and MGLI was more severe after wet-heating than after dry-heating regardless of the level of the seed maturity. Significantly positive correlations (*p* < 0.001) between the contents of MGNI, MDZI, and MGLI individually and of TIMG were observed in all the samples (Table 4). The contents of the three types of malonyl isoflavone in the immature soybeans did not change after 30 min of dry-heating but drastically decreased after 30 min of wet-heating. Similar results have also been observed by Chien et al. [34], where moist-heating at 100 °C reduced the MGNI (standard compound) content more than dry-heating at 100 °C.

Of the acetylglycoside isoflavones, ADZI (5.20 mg/100 g DW in immature soybeans; 3.09 mg/100 g DW in mature soybeans) was the main acetyl isoflavone in the FD samples (Table 2 and Table 3). Only a trace amount of AGNI was detected in both the immature and mature soybeans, with no AGLI being detected. The three types of acetyl isoflavone in the soybeans of different maturity levels responded differently to thermal processing. The ADZI content changed relatively little in the dry-heated immature soybeans but increased by four to five times in the dry-heated mature soybeans. The ADZI content also decreased to undetectable levels in the wet-heated immature soybeans, but not in the wet-heated mature soybeans. More AGLI and AGNI were produced in the dry-heated soybeans regardless of the maturity level. The AGLI content of the immature soybeans did not change but increased slightly after wet-heating. The AGNI in wet-heating was a key molecule because its content was unchanged in the immature soybeans after processing but increased in the mature soybeans, thus determining the amount of total acetylglycosides after wet-heating. The increase in the AGNI content was greater than that in the contents of ADZI and AGLI, possibly because of the varying thermal stability of the three acetylglycoside isoflavones and the corresponding malonylglycoside isoflavones. In contrast, the content of most of the types of acetyl increased up to 90 to 120 min of dry-heating regardless of the seed maturity. Significant positive correlations were found between the contents of ADZI, AGLI, AGNI, and that of TIAG for all the treatment groups except for the wet-heated immature soybeans (Table 4). A high negative correlation between the contents of AGNI and TIAG was observed for wet-heated immature soybeans, unlike the significant positive correlations for the other samples. This indicated that the amount of total acetylglycosides depended mainly on the content of AGNI during thermal processing.

The β-glycosides, the non-conjugated form of isoflavones, are the second major group after malonylglycosides in raw soybeans [52,53]. Of the β-glycosides, the content of GLI (11.62 mg/100 g DW) was higher than that of DZI (3.71 mg/100 g DW) and GNI (8.08 mg/100 g DW) in the FD immature soybeans (Table 2), similar to results from Simonne et al. [38]. The content of GLI (7.53 mg/100 g DW) was relatively lower than that of DZI (7.56 mg/100 g DW) and GNI (8.64 mg/100 g DW) in the FD mature soybeans (Table 3), results that are consistent with those of Kim et al. [54]. During thermal processing, different patterns of variation in the contents of GLI, GNI, and DZI arose. In the mature soybeans, the increase in the GLI content was small after heating compared with a significant increase in the DZI and GNI contents, similar to results reported by Toda et al. [48]. In the immature soybeans, no significant differences (*p* > 0.05) in GLI content were observed between FD and thermally treated soybeans. The contents of DZI and GNI both increased significantly after thermal treatment regardless of the seed maturity and processing method. Wet-heating was also more efficient in increasing β-glycoside isoflavones than dry-heating. The contents of GNI and DZI of all the treated samples were significantly positively correlated (*p* < 0.001) with the TIG content (Table 4). A good correlation (*p* < 0.01) between the GLI and TIG contents during thermal processing was found in the mature soybeans but not in the immature soybeans (*p* > 0.05). This indicated that the patterns of variation in the TIG content of soybeans were dominated more by the contents of DZI and GNI than the content of GLI during thermal processing even though the GLI content was relatively high in both FD samples.

In the FD soybeans, only small amounts of DZE (immature soybeans, 0.77 mg/100 g DW; mature soybeans, 1.21 mg/100 g DW) and GNE (immature soybeans, 0.22 mg/100 g DW; mature seeds, 0.94 mg/100 g DW) were detected, with a trace amount of GLE. The contents of the three aglycones in the immature soybeans were relatively stable under dry-heating but decreased to undetectable levels after 90 min of wet-heating. Both types of thermal processing decreased the contents of the three aglycones in the mature soybeans, particularly wet-heating. The variations in the contents of the three aglycones with the temperatures of thermal processing have been contradictory: Xu et al. [52] reported that aglycones in soybean flour extracts were generated with heat treatments above 135 °C, but Huang and Chou [35] reported that the contents of GNE, DZE, and GLE in black soybeans decreased at steaming temperatures of 60 °C or above for 30 min. A good correlation between the contents of DZE and TIA was found in all the treatments except for the dry-heated immature soybeans (Table 4). However, only the GLE content was well correlated with the TIA content in the immature soybeans under dry-heating with the content of GNE being well correlated with the TIA content in the mature soybeans under wet-heating.

The three isoflavone types (daidzein, glycitein, and genistein) showed different conversion patterns under heat processing. The MDZI and MGNI decreased more rapidly in the initial 30 min than the MGLI. Moreover, the production of AGNI and GNI by heat processing were higher than that of other isoflavone types. Glycitein conjugate types had relatively low thermal-change compared to daidzein and genistein types regardless of the seed maturity. Similar results have been reported by Stintzing et al. [55], where glycitein carrying a meth-oxy group at 6 position of A-ring has higher stability upon dry-heating. Moreover, Mathias et al. [56] reported that the heat-induced loss of daidzein glycosides was higher than that of genistein glycosides. These results indicate that different deglycosylation rates among isoflavone types occur during different thermal process methods.

### 2.4. Verification of the Relationship between Soybean Water Content and Changes in Patterns of Isoflavone Contents

It is important to note the different patterns of variation in the contents of acetylglycoside and β-glycoside isoflavones during the dry- and wet-heating of soybeans at two maturity levels. The internal moisture content of the soybeans affected the composition of isoflavones during thermal processing. To confirm this assumption, fully mature soybeans were soaked in distilled water for 0, 1, 2, 4, and 8 h to obtain different internal water contents, then dry-heated, followed by further observations of the patterns of variation in isoflavone content after heating for 1 h. Figure 4A shows the variations in the moisture content of the soybeans after soaking. The water content of the soybeans gradually increased from 6.34% to 57.04% as the soaking time increased. The contents of TI and of the four forms of isoflavone, TMIG, TAIG, TIG, and TIA, in the fully mature soybeans before and after the 1-h dry-heat treatment are shown in Figure 4B, C, D, E, and F, respectively.

Before heating, no significant differences in the TI content were observed between the unsoaked and soaked soybeans. Wang and Murphy [24] have also reported that soaking at room temperature for 10 to 12 h significantly increased the moisture content from 11.03% to 63.23% and retained the TI of the soaked soybeans. Heating significantly decreased the content of TI and TIMG and increased the content of TIAG and TIG of the soybeans compared with the FD samples (Figure 4B–E). The highest amount of TIAG generated was found in the unsoaked soybeans (0 h), and the lowest amount in soybeans soaked for a long time. There were no significant variations in the TIG content between the unsoaked and soaked soybeans after dry-heating for 1 h. These results were consistent with this report that the immature and mature beans had a similar TIG content after a long period of dry-heating, with even immature soybeans showing a lower TIAG content than mature soybeans (Figure 2). In contrast, the TIA content of the unsoaked soybeans was reduced by dry-heating, but, the longer the soaking time, the more TIA was generated after heating (Figure 4F). These results were different from the results we reported before, which may have been caused by differences between natural soybeans with a higher internal water content and artificially made soybeans with a higher water content. This also confirmed the assumption that the internal moisture content of soybeans was an important factor affecting the different patterns of variation in isoflavone content in soybeans of different maturity.

It is notable that, the longer the soaking time, the less TIAG was produced after heating. Lee and Lee [46] reported that the content of acetyl isoflavones in soybeans soaked for 12 h did not change during 120 min of oven drying but that, in unsoaked soybeans, it increased significantly after roasting at 200 °C. This indicated that soybeans with a higher internal water content produced a lower amount of acetyl isoflavones after heating, which confirmed the previous assumption that the water content of soybeans significantly affected the pattern of isoflavone conversion. Therefore, the differences between the content of acetyl isoflavones in mature and immature soybeans after heating were caused by the difference in the internal water content. The increase in the content of aglycone isoflavones was highly related to soaking and heating, results similar to those of Lima et al. [57], who found no significant difference in the content of aglycones in soybeans soaked at 25 °C but a significant increase after soaking for 1 h at 70 °C.

## 3. Materials and Methods

### 3.1. Chemical Reagents

The HPLC-grade acetonitrile and water (Daejung Chemical & Metals Co., Siheung, Korea) were used as mobile phases for isoflavones analysis. Standards of isoflavone aglycones (daidzein, glycitein, and genistein) and β-glycosides (daidzin, glycitin, and genistin) were purchased from LC Laboratories (Woburn, MA, USA). Isoflavone acetylglycosides (acetyldaidzin, acetylglycitin, and acetylgenistin) were obtained from Nacalai tesque (Kyoto, Japan), and malonylglycosides (malonyldaidzin, malonylglycitin, and malonylgenistin) were obtained from GenDEPOT (Katy, TX, USA).

### 3.2. Soybean Cultivation

Soybean seeds (*Glycine max* L. cv. *Pungwon*) used in this study were provided by the Pulmuone Food Co. (Chungbuk, Korea). The cultivar ‘Pungwon’ was registered to the Korea Seed & Variety Service (Gimcheon, Korea) in 2007 and had earlier matu-ration period and high content of isoflavones (more information described in Oh et al. [58]). The soybeans were germinated for 24 h at room temperature in a dark culture room after soaking with distilled water for 4 h. The germinated soybeans were planted in a horticultural soil (Baroker, Seoulbio Co., Eumseong, Korea) in pots (Plastic pot, 24 × 27 × 18 cm) in early June 2020 and then grown in the greenhouse of Kyung Hee University (Yongin, Korea) under natural sunlight. The average of temperature during the soybean growing season was 18–22 °C in June; 22–30 °C in July and August; 19–26 °C in September; 12–23 °C in October (based on Korean meteorological administration data). The average solar radiation period was 14 h/day in June to August and 12.5 h/day in September and October (based on Korean meteorological administration data). The average of relative humidity was 45–55% from June to October. The potted soybeans were maintained with several irrigations per week in the early stage of soybean plants and with daily irrigation in the period of seed formation. The soybean seeds were harvested at the immature stage on September 10 when the pods of soybeans contained green seeds that filled the pod cavity and harvested at the mature stage on October 10 when 95% of the pods exhibited the light brown color with dehydrating, as shown in Figure 1 in a previous report [36].

### 3.3. Physical Characteristics of Immature and Mature Seeds

The harvested soybean samples were weighed before and after freeze-drying. The dry weight of the immature and mature soybeans was expressed as the weight (g) of 10 raw seeds based on the mean value of ten replicates. The water content (%) was calculated as follows: 100 × [fresh weight (g) − dry weight (g)]/[fresh weight (g)]. Fresh soybeans (0.3 g) were ground with a pestle and a mortar and added 0.6 mL of distilled water to measure the soluble solid content (SSC). After stirring the mixture, the sample was centrifuged at 14,240× *g* for 15 min. The SSC of the supernatant was evaluated using a hand refractometer (Atago Co., Tokyo, Japan) and expressed as degree of Brix (°Brix).

### 3.4. Thermal Treatment

The immature and mature soybean seeds were processed using three thermal processing methods: (1) freeze-drying (FD) at −80 °C for 72 h in a vacuum freeze-dryer (IlshinBioBase. Co. Ltd., Dongducheon, Korea) and stored in a −20 °C refrigerator; (2) dry-heating at 100 ± 3 °C for 30, 60, 90, and 120 min with a convective dryer (Koencon Co., Ltd., Hanam, Korea); and (3) wet-heating (steaming) at 100 ± 3 °C for 30, 60, 90, and 120 min with a steam cooker. All experiments were carried out in triplicate. The thermally treated samples were freeze-dried and stored in a −20 °C refrigerator before isoflavone analysis.

### 3.5. Extraction of Isoflavones

All samples were finely ground using a commercial grinder (JL-1000, Hibell, Hwaseong, Korea). The isoflavones extraction was performed by previously described method [59]. Briefly, 20 mg of ground sample mixed with 58% aqueous acetonitrile (1 mL, *v*/*v*) in a shaking incubator for 24 h at 25 °C and 120 rpm after sonication for 30 min. The supernatant was obtained after centrifuging at 14,240× *g* for 5 min. Then, two-fold volume of distilled water was added to dilute the supernatant. The diluted supernatant was filtered through a 0.45 μm hydrophilic PTFE membrane syringe filter (Futecs Co., Ltd., Daejeon, Korea) and used for isoflavones analysis.

### 3.6. Determination of Isoflavones

Extracts were analyzed using HPLC (Waters 2695 Alliance HPLC; Waters Inc., Milford, MA, USA) with the octadecylsilane column (Prontosil 120–5-C18-SH-EPS 5.0 μm (200 × 4.6 mm; Bischoff, Leonberg, Germany). According to the previously published method [59], the solvent A (0.1% formic acid in water) and solvent B (0.1% formic acid in acetonitrile) were used as mobile phase with the flow rate of 0.8 mL/min. The mobile phase B gradient was as follows: 16–25%, 0 to 35 min; 25–50%, 35 to 40 min; 50–65%, 40 to 47 min; 65–16%, 47 to 50 min. The injection volume was 5 μL. The peaks of 12 standard isoflavones were detected at 254 nm (Water 996 photodiode array detector (Waters Inc.)).

### 3.7. Statistical Analysis

All experiments were carried out in triplicate with the data expressed as the mean with standard error (*n* = 3). Analysis of variance was performed using SAS software (Enterprise guide 7.1 version, SAS Institute Inc., Cary, NC, USA). Significant differences between experimental treatments were evaluated using Tukey’s student range test, with a significance level defined at *p* < 0.05.

## 4. Conclusions

This is the first study to compare the patterns of isoflavone changes between soybeans at two maturity levels after thermal processing. Overall, the patterns of the isoflavone changes in the soybeans depended significantly on the soybean maturity and the processing method, which affected the decarboxylation of malonylglycoside or the deesterification of acetylglycoside isoflavones. The decreases in the TI in all the samples were mainly caused by the decrease in the TIMG during thermal processing. The deglycosylation of the three types of malonyl isoflavones was more severe in wet- than in dry-heating regardless of the seed maturity. In the acetylglycoside isoflavones, dry-heating produced a relatively low amount of acetyl isoflavones in the immature seeds compared with that in the mature seeds. The ADZI was relatively less changed in the dry-heated immature seeds but increased significantly in the processed mature seeds. AGLI and AGNI were produced in greater amounts in the dry-heated samples regardless of the seed maturity. The AGNI in wet-heating was the key molecule because its content remained unchanged in the immature soybeans during processing but increased in the mature soybeans, which determined the total amount of acetylglycoside in wet-heating. Wet-heating increased the amount of β-glycoside isoflavones in the mature soybeans more than in dry-heating, while, interestingly, the immature soybeans exhibited the opposite behavior. The aglycone isoflavones were stable under dry-heating, but their contents decreased significantly after wet-heating. The internal moisture content of the soybeans was an important factor affecting the deglycosylation of isoflavones during thermal processing, also confirmed by the verification experiment (Section 2.4). This is the first study to highlight the importance of the internal water content of soybeans on the distribution of isoflavones during thermal processing. The results of the present study will provide basic information on the different uses of immature and mature soybeans after thermal processing.

## Figures and Tables

**Table 1:** Physical characteristics of immature and mature soybean seeds.

Seed Maturity	Water Content (%)	Dry Weight (g/10 ea)	SSC (°Brix)
Immature	66.87 ± 0.32 ^a^	0.66 ± 0.02 ^b^	2.75 ± 0.18 ^b^
Mature	13.04 ± 0.22 ^b^	0.88 ± 0.03 ^a^	15.76 ± 0.31 ^a^

SSC means soluble solids content. Different letters (a, b) in a column indicate significant differences at *p* < 0.05 by Tukey’s studentized range (HSD) test. Results are given as mean ± SE (*n* = 10).

**Table 2:** Isoflavone content (mg/100 g DW) in immature soybeans during thermal processing.

		FD (0 Min)	Dry-Heating				Wet-Heating			
			30 Min	60 Min	90 Min	120 Min	30 Min	60 Min	90 Min	120 Min
Malonyl glycosides	MDZI	62.32 ± 1.54 ^a^	63.38 ± 9.37 ^a^	34.81 ± 3.21 ^b^	29.62 ± 3.55 ^b^	23.01 ± 2.31 ^b,c^	23.16 ± 2.64 ^b,c^	9.39 ± 1.27 ^c,d^	4.01 ± 0.08 ^d^	2.03 ± 0.34 ^d^
	MGLI	29.26 ± 2.47 ^a^	26.09 ± 7.54 ^a^	18.51 ± 3.35 ^a,b^	10.51 ± 1.80 ^b,c^	7.70 ± 0.63 ^b,c^	5.73 ± 0.42 ^c^	1.57 ± 0.44 ^c^	0.62 ± 0.21 ^c^	N.D.
	MGNI	78.18 ± 0.21 ^a^	72.87 ± 9.58 ^a^	57.24 ± 4.00 ^a,b^	40.79 ± 6.04 ^b,c^	37.21 ± 4.76 ^b,c^	38.68 ± 2.06 ^b,c^	20.07 ± 1.80 ^c,d^	7.13 ± 0.83 ^d^	4.23 ± 0.48 ^d^
Acetyl glycosides	ADZI	5.20 ± 0.29 ^a^	2.80 ± 0.62 ^b,c^	2.64 ± 0.40 ^b,c^	4.39 ± 0.70 ^a,b^	5.66 ± 0.70 ^a^	1.06 ± 0.20 ^c^	0.68 ± 0.05 ^c^	N.D.	N.D.
	AGLI	N.D.	Tr.	0.36 ± 0.19 ^a^	2.56 ± 0.77 ^a^	3.65 ± 1.23 ^a^	N.D.	N.D.	N.D.	N.D.
	AGNI	Tr.	0.12 ± 0.12 ^c,d^	2.20 ± 0.26 ^c,d^	4.02 ± 1.83 ^b^	9.94 ± 1.27 ^a^	2.25 ± 0.25 ^c,d^	2.86 ± 0.13 ^c^	2.25 ± 0.17 ^c,d^	2.04 ± 0.16 ^c,d^
β-glycosides	DZI	3.71 ± 0.53 ^c^	5.16 ± 1.17 ^b,c^	6.23 ± 0.59 ^b,c^	10.11 ± 2.81 ^a,b,c^	15.45 ± 1.80 ^a^	8.91 ± 1.47 ^a,b,c^	11.72 ± 1.51 ^a,b^	10.55 ± 1.02 ^a,b,c^	9.11 ± 0.70 ^a,b,c^
	GLI	11.62 ± 1.16 ^a^	11.30 ± 3.98 ^a^	10.82 ± 1.37 ^a^	10.84 ± 2.27 ^a^	11.70 ± 1.50 ^a^	8.62 ± 1.08 ^a^	7.42 ± 1.12 ^a^	6.24 ± 0.80 ^a^	7.31 ± 0.48 ^a^
	GNI	8.08 ± 0.96 ^e^	10.26 ± 1.90 ^d,e^	14.30 ± 0.57 ^c,d,e^	22.52 ± 2.20 ^a,b,c^	30.16 ± 2.90 ^a^	19.16 ± 1.80 ^b,c,d^	26.12 ± 2.22 ^a,b^	25.47 ± 2.30 ^a,b^	19.9 ± 1.41 ^b,c^
Aglycones	DZE	0.77 ± 0.12 ^a^	0.33 ± 0.03 ^a^	N.D.	0.61 ± 0.24 ^a^	0.75 ± 0.32 ^a^	N.D.	N.D.	N.D.	N.D.
	GLE	Tr.	0.28 ± 0.14 ^a^	0.74 ± 0.42 ^a^	1.13 ± 0.25 ^a^	1.03 ± 0.42 ^a^	N.D.	N.D.	N.D.	N.D.
	GNE	0.22 ± 0.11 ^a,b^	0.70 ± 0.12 ^a,b^	0.74 ± 0.17 ^a,b^	0.87 ± 0.21 ^a^	0.89 ± 0.14 ^a^	0.07 ± 0.07 ^b^	0.22 ± 0.14 ^a,b^	N.D.	N.D.

Data are shown as mean with standard error (*n* = 3). Different letters (a–e) in a row indicate significant differences at *p* < 0.05 by Tukey’s studentized range (HSD) test. N.D. indicates not detected; Tr. Indicates trace amount.

**Table 3:** Isoflavone content (mg/100 g DW) in mature soybeans during thermal processing.

		FD (0 Min)	Dry-Heating				Wet-Heating			
			30 Min	60 Min	90 Min	120 Min	30 Min	60 Min	90 Min	120 Min
Malonyl glycosides	MDZI	119.20 ± 11.24 ^a^	68.96 ± 4.43 ^b^	57.43 ± 3.54 ^b,c^	47.09 ± 2.40 ^b,c,d^	31.92 ± 1.20 ^d,e^	48.00 ± 2.54 ^b,c,d^	35.03 ± 1.36 ^c,d,e^	20.91 ± 0.64 ^d,e^	18.34 ± 1.20 ^e^
	MGLI	24.18 ± 5.96 ^a^	21.53 ± 1.52 ^a^	22.27 ± 2.22 ^a,b^	15.58 ± 2.61 ^b,c^	14.15 ± 1.38 ^b,c^	16.22 ± 2.80 ^b,c^	12.87 ± 2.84 ^c^	4.65 ± 0.28 ^c^	4.09 ± 0.22 ^c^
	MGNI	141.25 ± 7.92 ^a^	89.11 ± 3.38 ^b^	80.17 ± 3.10 ^b^	68.91 ± 2.22 ^b,c,d^	52.48 ± 3.44 ^d,e^	63.58 ± 0.59 ^b,c^	52.53 ± 0.48 ^c,d^	32.76 ± 1.20 ^e^	26.17 ± 0.76 ^e^
Acetyl glycosides	ADZI	3.09 ± 0.35 ^d^	5.74 ± 0.56 ^d^	10.46 ± 0.61 ^b,c^	15.83 ± 1.97 ^a^	12.23 ± 0.98 ^a,b^	5.96 ± 0.29 ^d^	5.65 ± 0.30 ^d^	6.32 ± 0.11 ^d^	6.81 ± 0.35 ^c,d^
	AGLI	N.D.	2.13 ± 0.83 ^c^	6.12 ± 1.48 ^b,c^	12.67 ± 1.04 ^a^	8.67 ± 2.17 ^a,b^	3.77 ± 0.66 ^b,c^	3.22 ± 0.65 ^b,c^	2.85 ± 0.25 ^c^	3.57 ± 0.66 ^b,c^
	AGNI	Tr.	5.62 ± 0.34 ^e^	14.51 ± 0.78 ^b^	21.10 ± 0.53 ^a^	20.58 ± 1.61 ^a^	6.57 ± 0.06 ^d,e^	9.09 ± 0.19 ^c,d^	9.42 ± 0.22 ^c,d^	11.84 ± 0.35 ^b,c^
β-glycosides	DZI	7.56 ± 0.80 ^e^	11.22 ± 0.66 ^d,e^	19.53 ± 1.11 ^b^	19.92 ± 0.61 ^b^	19.07 ± 0.88 ^b^	13.62 ± 1.00 ^c,d^	17.97 ± 0.58 ^b,c^	20.03 ± 0.58 ^b^	27.45 ± 0.38 ^a^
	GLI	7.53 ± 1.96 ^a,b^	7.71 ± 0.86 ^b^	15.05 ± 0.43 ^a,b^	14.47 ± 1.09 ^a,b^	10.37 ± 0.98 ^a,b^	14.82 ± 1.82 ^a,b^	17.30 ± 2.20 ^a^	13.97 ± 0.97 ^a,b^	16.75 ± 0.57 ^a^
	GNI	8.64 ± 1.99 ^e^	16.83 ± 0.58 ^d^	29.26 ± 1.04 ^c^	33.99 ± 0.87 ^b,c^	31.11 ± 2.08 ^b,c^	21.07 ± 0.18 ^d^	30.52 ± 0.71 ^b,c^	36.74 ± 0.91 ^b^	47.44 ± 1.62 ^a^
Aglycones	DZE	1.21 ± 0.35 ^a^	0.50 ± 0.10 ^a,b^	0.78 ± 0.11 ^a,b^	0.63 ± 0.05 ^a,b^	0.59 ± 0.09 ^a,b^	0.38 ± 0.03 ^b^	0.29 ± 0.15 ^b^	0.31 ± 0.16 ^b^	0.57 ± 0.13 ^a,b^
	GLE	Tr.	N.D.	N.D.	N.D.	N.D.	0.38 ± 0.02 ^a^	N.D.	N.D.	N.D.
	GNE	0.94 ± 0.14 ^a^	0.61 ± 0.08 ^a,b,c^	0.70 ± 0.07 ^a,b,c^	0.90 ± 0.12 ^a^	0.72 ± 0.04 ^a,b^	0.27 ± 0.06 ^c^	0.30 ± 0.02 ^b,c^	0.40 ± 0.10 ^b,c^	0.45 ± 0.10 ^b,c^

Data are shown as mean with standard error (*n* = 3). Different letters (a–e) next to data in a row indicate significant differences at *p* < 0.05 by Tukey’s studentized range (HSD) test. N.D. indicates not detected; Tr. Indicates trace amount.

**Table 4:** Correlation coefficients between isoflavone forms and individual isoflavones in thermally treated soybeans.

Seed Maturation	Heating Methods		MDZI	MGLI	MGNI
Immature	D ^z^	TIMG	0.983 ***	0.931 ***	0.970 ***
	W ^y^	TIMG	0.999 ***	0.976 ***	0.944 ***
Mature	D	TIMG	0.996 ***	0.712 ***	0.987 ***
	W	TIMG	0.996 ***	0.889 ***	0.997 ***
			**ADZI**	**AGLI**	**AGNI**
Immature	D	TIAG	0.704 **	0.963 ***	0.967 ***
	W	TIAG	0.902 ***	-	−0.537 *
Mature	D	TIAG	0.961 ***	0.955 ***	0.980 ***
	W	TIAG	0.901 **	0.844 ***	0.983 ***
			**DZI**	**GLI**	**GNI**
Immature	D	TIG	0.939 ***	0.410 ^ns^	0.946 ***
	W	TIG	0.972 ***	−0.314 ^ns^	0.953 ***
Mature	D	TIG	0.943 ***	0.769 ***	0.956 ***
	W	TIG	0.983 ***	0.687 **	0.976 ***
			**DZE**	**GLE**	**GNE**
Immature	D	TIA	−0.053 ^ns^	0.892 ***	0.420 ^ns^
	W	TIA	1.000 ***	-	-
Mature	D	TIA	0.601 *	-	0.202 ^ns^
	W	TIA	0.727 **	-	0.658 **

^z^ and ^y^ indicate dry-heating and wet-heating, respectively. *, **, and *** indicate significances at *p* < 0.05, *p* < 0.01, and *p* < 0.001 in Tukey’s HSD test. ^ns^ indicates no significance at the test. Data of 12 individual isoflavones, TIMG, TIAG, TIG, and TIA, for correlation analysis were calculated by the time-dependent dry-heating and wet-heating values, ranging from 0 to 120 min treatments.

## Data Availability

The data presented in this study are available on request from the corresponding author.

## Sample Availability

Samples of the compounds are not available from the authors.
